# Supplementary material for: Protein synthesis inhibition and loss of homeostatic functions in astrocytes from an Alzheimer’s disease mouse model: a role for ER-mitochondria interaction
Source: Cell Death Dis. 2022 Oct 18;13(10):878. doi: 10.1038/s41419-022-05324-4 (PMC9579125; doi:10.1038/s41419-022-05324-4)
Supplement: Supplementary file 3 — Supplemental table 1 [file 41419_2022_5324_MOESM3_ESM.pdf]

Supplemental Table 1.

Shotgun mass spectrometry proteomics of ACM from WT-iAstro and 3Tg-iAstro cells

Identified proteins

N = 3 independent cultures for each genotype

Uniptor_ID	Uniprot_KB	Description	Gene name	Identified in WT, 3Tg or both
SYCP1_MOUSE	Q62209	Synaptonemal complex protein 1 (SCP-1)	Sycp1 Scp1	WT- and 3Tg-iAstro cells
KPYM_MOUSE	P52480	Pyruvate kinase PKM (EC 2.7.1.40) (Pyruvate kinase muscle isozyme)	Pkm Pk3 Pkm2 Pykm	WT- and 3Tg-iAstro cells
PPIA_MOUSE	P17742	Peptidyl-prolyl cis-trans isomerase A (PPIase A) (EC 5.2.1.8) (Cyclophilin A) (Cyclosporin A-binding protein) (Rotamase A) (SP18) [Cleaved into: Peptidyl-prolyl cis-trans isomerase A, N-terminally processed]	Ppia	WT- and 3Tg-iAstro cells
FSTL1_MOUSE	Q62356	Follistatin-related protein 1 (Follistatin-like protein 1) (TGF-beta-inducible protein TSC-36)	Fstl1 Frp Fstl Tsc36	WT- and 3Tg-iAstro cells
VIME_MOUSE	P20152	Vimentin	Vim	WT- and 3Tg-iAstro cells
HS90B_MOUSE	P11499	Heat shock protein HSP 90-beta (Heat shock 84 kDa) (HSP 84) (HSP84) (Tumor-specific transplantation 84 kDa antigen) (TSTA)	Hsp90ab1 Hsp84 Hsp84-1 Hspcb	WT- and 3Tg-iAstro cells
NRK_MOUSE	Q9R0G8	Nik-related protein kinase (EC 2.7.11.1) (Nck-interacting kinase-like embryo specific kinase) (NESK) (NIK-like embryo-specific kinase)	Nrk Nesk	WT- and 3Tg-iAstro cells
ALBU_MOUSE	P07724	Albumin	Alb Alb-1 Alb1	WT- and 3Tg-iAstro cells

OSTP_MOUSE	P10923	Osteopontin (2AR) (Bone sialoprotein 1) (Calcium oxalate crystal growth inhibitor protein) (Early T-lymphocyte activation 1 protein) (Minopontin) (Secreted phosphoprotein 1) (SPP-1)	Spp1 Eta-1 Op Spp-1	WT- and 3Tg-iAstro cells
AFAM_MOUSE	O89020	Afamin (Alpha-albumin) (Alpha-Alb)	Afm	WT- and 3Tg-iAstro cells
CATD_MOUSE	P18242	Cathepsin D (EC 3.4.23.5)	Ctsd	WT- and 3Tg-iAstro cells
K2C75_MOUSE	Q8BGZ7	Keratin, type II cytoskeletal 75 (Cytokeratin-75) (CK-75) (Keratin-6 hair follicle) (mK6hf) (Keratin-75) (K75) (Type II keratin-K6hf) (Type-II keratin Kb18)	Krt75 Kb18	WT- and 3Tg-iAstro cells
A2MG_MOUSE	Q6GQT1	Alpha-2-macroglobulin-P (Alpha-2-macroglobulin)	A2m A2mp	WT- and 3Tg-iAstro cells
NRAP_MOUSE	Q80XB4	Nebulin-related-anchoring protein (N-RAP)	Nrap	WT- and 3Tg-iAstro cells
K22O_MOUSE	Q3UV17	Keratin, type II cytoskeletal 2 oral (Keratin-76) (K76) (Type-II keratin Kb9)	Krt76	WT- and 3Tg-iAstro cells
ITIH3_MOUSE	Q61704	Inter-alpha-trypsin inhibitor heavy chain H3 (ITI heavy chain H3) (ITI-HC3) (Inter-alpha-inhibitor heavy chain 3)	Itih3	WT- and 3Tg-iAstro cells
SPRC_MOUSE	P07214	SPARC (Basement-membrane protein 40) (BM-40) (Osteonectin) (ON) (Secreted protein acidic and rich in cysteine)	Sparc	WT- and 3Tg-iAstro cells
VNN3_MOUSE	Q9QZ25	Vascular non-inflammatory molecule 3 (Vanin-3) (EC 3.5.1.92)	Vnn3	WT- and 3Tg-iAstro cells
CBPE_MOUSE	Q00493	Carboxypeptidase E (CPE) (EC 3.4.17.10) (Carboxypeptidase H) (CPH) (Enkephalin convertase) (Prohormone-processing carboxypeptidase)	Cpe	WT- and 3Tg-iAstro cells

FA92A_MOUSE	Q8BP22	Protein FAM92A	Fam92a Fam92a1	WT- and 3Tg-iAstro cells
RC3H2_MOUSE	P0C090	Roquin-2 (EC 2.3.2.27) (Membrane-associated nucleic acid-binding protein) (RING finger and CCCH-type zinc finger domain-containing protein 2) (RING-type E3 ubiquitin transferase Roquin-2)	Rc3h2 Mnab	WT- and 3Tg-iAstro cells
FINC_MOUSE	P11276	Fibronectin (FN) [Cleaved into: Anastellin]	Fn1	WT- and 3Tg-iAstro cells
TBA1B_MOUSE	P05213	Tubulin alpha-1B chain (Alpha-tubulin 2) (Alpha-tubulin isotype M-alpha-2) (Tubulin alpha-2 chain) [Cleaved into: Detyrosinated tubulin alpha-1B chain]	Tuba1b Tuba2	WT- and 3Tg-iAstro cells
TTHY_MOUSE	P07309	Transthyretin (Prealbumin)	Ttr	WT- and 3Tg-iAstro cells
FBLN1_MOUSE	Q08879	Fibulin-1 (FIBL-1) (Basement-membrane protein 90) (BM-90)	Fbln1	WT- and 3Tg-iAstro cells
ITIH4_MOUSE	A6X935	Inter alpha-trypsin inhibitor, heavy chain 4 (ITI heavy chain H4) (ITI-HC4) (Inter-alpha-inhibitor heavy chain 4)	Itih4	WT- and 3Tg-iAstro cells
SAP_MOUSE	Q61207	Prosaposin (Sulfated glycoprotein 1) (SGP-1) [Cleaved into: Saposin-A; Saposin-B-Val; Saposin-B; Saposin-C; Saposin-D]	Psap Sgp1	WT- and 3Tg-iAstro cells
ANT3_MOUSE	P32261	Antithrombin-III (ATIII) (Serpin C1)	Serpinc1 At3	WT- and 3Tg-iAstro cells
NUCB1_MOUSE	Q02819	Nucleobindin-1 (CALNUC)	Nucb1 Nuc Nucb	WT- and 3Tg-iAstro cells
K2C79_MOUSE	Q8VED5	Keratin, type II cytoskeletal 79 (Cytokeratin-79) (CK-79) (Keratin-79) (K79) (Type-II keratin Kb38)	Krt79 Kb38	WT- and 3Tg-iAstro cells

K1C10_MOUSE	P02535	Keratin, type I cytoskeletal 10 (56 kDa cytokeratin) (Cytokeratin-10) (CK-10) (Keratin, type I cytoskeletal 59 kDa) (Keratin-10) (K10)	Krt10 Krt1-10	WT- and 3Tg-iAstro cells
TRFE_MOUSE	Q921I1	Serotransferrin (Transferrin) (Beta-1 metal-binding globulin) (Siderophilin)	Tf Trf	WT- and 3Tg-iAstro cells
DYST_MOUSE	Q91ZU6	Dystonin (Bullous pemphigoid antigen 1) (BPA) (Dystonia musculorum protein) (Hemidesmosomal plaque protein) (Microtubule actin cross-linking factor 2)	Dst Bpag1 Macf2	WT- and 3Tg-iAstro cells
CO3_MOUSE	P01027	Complement C3 (HSE-MSF) [Cleaved into: Complement C3 beta chain; C3-beta-c (C3bc); Complement C3 alpha chain; C3a anaphylatoxin; Acylation stimulating protein (ASP) (C3adesArg); Complement C3b alpha' chain; Complement C3c alpha' chain fragment 1; Complement C3dg fragment; Complement C3g fragment; Complement C3d fragment; Complement C3f fragment; Complement C3c alpha' chain fragment 2]	C3	WT- and 3Tg-iAstro cells
IBP2_MOUSE	P47877	Insulin-like growth factor-binding protein 2 (IBP-2) (IGF-binding protein 2) (IGFBP-2) (mIGFBP-2)	Igfbp2 Igfbp-2	WT- and 3Tg-iAstro cells
ARAP3_MOUSE	Q8R5G7	Arf-GAP with Rho-GAP domain, ANK repeat and PH domain-containing protein 3 (Centaurin-delta-3) (Cnt-d3) (Dual specificity Rho- and Arf-GTPase-activating protein 1)	Arap3 Centd3 Drag1 Kiaa4097	WT- and 3Tg-iAstro cells

FETUA_MOUSE	P29699	Alpha-2-HS-glycoprotein (Countertrypin) (Fetuin-A)	Ahsg Fetua	WT- and 3Tg-iAstro cells
CO4B_MOUSE	P01029	Complement C4-B [Cleaved into: Complement C4 beta chain; Complement C4 alpha chain; C4a anaphylatoxin; Complement C4 gamma chain]	C4b C4	WT- and 3Tg-iAstro cells
LUM_MOUSE	P51885	Lumican (Keratan sulfate proteoglycan lumican) (KSPG lumican)	Lum Lcn Ldc	WT- and 3Tg-iAstro cells
HBB2_MOUSE	P02089	Hemoglobin subunit beta-2 (Beta-2-globin) (Hemoglobin beta-2 chain) (Hemoglobin beta-minor chain)	Hbb-b2	WT- and 3Tg-iAstro cells
TPM4_MOUSE	Q6IRU2	Tropomyosin alpha-4 chain (Tropomyosin-4)	Tpm4	WT- and 3Tg-iAstro cells
TENA_MOUSE	Q80YX1	Tenascin (TN) (Hexabrachion) (Tenascin-C) (TN-C)	Tnc Hxb	WT- and 3Tg-iAstro cells
TSP1_MOUSE	P35441	Thrombospondin-1 (Glycoprotein G)	Thbs1 Tsp1	WT- and 3Tg-iAstro cells
PZP_MOUSE	Q61838	Pregnancy zone protein (Alpha-2-macroglobulin) (Alpha-2-M) [Cleaved into: Alpha-2-macroglobulin 165 kDa subunit; Alpha-2-macroglobulin 35 kDa subunit]	Pzp A2m	WT- and 3Tg-iAstro cells
PLMN_MOUSE	P20918	Plasminogen (EC 3.4.21.7) [Cleaved into: Plasmin heavy chain A; Activation peptide; Angiostatin; Plasmin heavy chain A, short form; Plasmin light chain B]	Plg	WT- and 3Tg-iAstro cells
K2C1B_MOUSE	Q6IFZ6	Keratin, type II cytoskeletal 1b (Cytokeratin-1B) (CK-1B) (Embryonic type II keratin-1) (Keratin-77) (K77) (Type-II keratin Kb39)	Krt77 Krt1b	WT- and 3Tg-iAstro cells

ACTB_MOUSE	P60710	Actin, cytoplasmic 1 (Beta-actin) [Cleaved into: Actin, cytoplasmic 1, N-terminally processed]	Actb	WT- and 3Tg-iAstro cells
APOA1_MOUSE	Q00623	Apolipoprotein A-I (Apo-AI) (ApoA-I) (Apolipoprotein A1) [Cleaved into: Proapolipoprotein A-I (ProapoA-I); Truncated apolipoprotein A-I]	Apoa1	WT- and 3Tg-iAstro cells
ITIH2_MOUSE	Q61703	Inter-alpha-trypsin inhibitor heavy chain H2 (ITI heavy chain H2) (ITI-HC2) (Inter-alpha-inhibitor heavy chain 2)	Itih2	WT- and 3Tg-iAstro cells
ACTG_MOUSE	P63260	Actin, cytoplasmic 2 (Gamma-actin) [Cleaved into: Actin, cytoplasmic 2, N-terminally processed]	Actg1 Actg	WT- and 3Tg-iAstro cells
K2C1_MOUSE	P04104	Keratin, type II cytoskeletal 1 (67 kDa cytokeratin) (Cytokeratin-1) (CK-1) (Keratin-1) (K1) (Type-II keratin Kb1)	Krt1 Krt2-1	WT- and 3Tg-iAstro cells
MUG1_MOUSE	P28665	Murinoglobulin-1 (MuG1)	Mug1 Mug-1	WT- and 3Tg-iAstro cells
KNG1_MOUSE	O08677	Kininogen-1 [Cleaved into: Kininogen-1 heavy chain; Bradykinin; Kininogen-1 light chain]	Kng1 Kng	WT- and 3Tg-iAstro cells
PEDF_MOUSE	P97298	Pigment epithelium-derived factor (PEDF) (Caspin) (Serpin F1) (Stromal cell-derived factor 3) (SDF-3)	Serpinf1 Pedf Sdf3	WT- and 3Tg-iAstro cells
RS27A_MOUSE	P62983	Ubiquitin-40S ribosomal protein S27a (Ubiquitin carboxyl extension protein 80) [Cleaved into: Ubiquitin; 40S ribosomal protein S27a]	Rps27a Uba80 Ubcep1	WT- and 3Tg-iAstro cells
ACTC_MOUSE	P68033	Actin, alpha cardiac muscle 1 (Alpha-cardiac actin) [Cleaved into: Actin, alpha cardiac muscle 1, intermediate form]	Actc1 Actc	Only in WT-iAstro cells

DCTP1_MOUSE	Q9QY93	dCTP pyrophosphatase 1 (EC 3.6.1.12) (Deoxycytidine-triphosphatase 1) (dCTPase 1) (RS21-C6)	Dctpp1 Tdrg-TL1	Only in WT-iAstro cells
HEXA_MOUSE	P29416	Beta-hexosaminidase subunit alpha (EC 3.2.1.52) (Beta-N-acetylhexosaminidase subunit alpha) (Hexosaminidase subunit A) (N-acetyl-beta-glucosaminidase subunit alpha)	Hexa	Only in WT-iAstro cells
1433B_MOUSE	Q9CQV8	14-3-3 protein beta/alpha (Protein kinase C inhibitor protein 1) (KCIP-1) [Cleaved into: 14-3-3 protein beta/alpha, N-terminally processed]	Ywhab	Only in WT-iAstro cells
LRP5_MOUSE	Q91VN0	Low-density lipoprotein receptor-related protein 5 (LRP-5) (Low-density lipoprotein receptor-related protein 7) (LRP-7)	Lrp5 Lr3 Lrp7	Only in WT-iAstro cells
NPC2_MOUSE	Q9Z0J0	NPC intracellular cholesterol transporter 2 (Epididymal secretory protein E1) (mE1) (Niemann Pick type C2 protein homolog)	Npc2	Only in WT-iAstro cells
RL40_MOUSE	P62984	Ubiquitin-60S ribosomal protein L40 (Ubiquitin A-52 residue ribosomal protein fusion product 1) [Cleaved into: Ubiquitin; 60S ribosomal protein L40 (CEP52)]	Uba52 Ubcep2	Only in WT-iAstro cells
HS90A_MOUSE	P07901	Heat shock protein HSP 90-alpha (EC 3.6.4.10) (Heat shock 86 kDa) (HSP 86) (HSP86) (Tumor-specific transplantation 86 kDa antigen) (TSTA)	Hsp90aa1 Hsp86 Hsp86-1 Hspca	Only in WT-iAstro cells
H3C_MOUSE	P02301	Histone H3.3C (Embryonic)	H3-5 Gm14384 H3f3c	Only in WT-iAstro cells

NDKB_MOUSE	Q01768	Nucleoside diphosphate kinase B (NDK B) (NDP kinase B) (EC 2.7.4.6) (Histidine protein kinase NDKB) (EC 2.7.13.3) (P18) (nm23-M2)	Nme2	Only in WT-iAstro cells
TPM1_MOUSE	P58771	Tropomyosin alpha-1 chain (Alpha-tropomyosin) (Tropomyosin-1)	Tpm1 Tpm-1 Tpma	Only in WT-iAstro cells
CO1A2_MOUSE	Q01149	Collagen alpha-2(I) chain (Alpha-2 type I collagen)	Col1a2 Cola2	Only in WT-iAstro cells
CRLF1_MOUSE	Q9JM58	Cytokine receptor-like factor 1 (Cytokine receptor-like molecule 3) (CRLM-3) (Cytokine-like factor 1) (CLF-1) (Novel cytokine receptor 6) (NR6)	Crlf1 Crlm3	Only in WT-iAstro cells
B2MG_MOUSE	P01887	Beta-2-microglobulin	B2m	Only in WT-iAstro cells
ACTN1_MOUSE	Q7TPR4	Alpha-actinin-1 (Alpha-actinin cytoskeletal isoform) (F-actin cross-linking protein) (Non-muscle alpha-actinin-1)	Actn1	Only in WT-iAstro cells
PRDX6_MOUSE	O08709	Peroxiredoxin-6 (EC 1.11.1.27) (1-Cys peroxiredoxin) (1-Cys PRX) (Acidic calcium-independent phospholipase A2) (aiPLA2) (EC 3.1.1.4) (Antioxidant protein 2) (Glutathione-dependent peroxiredoxin) (Lysophosphatidylcholine acyltransferase 5) (LPC acyltransferase 5) (LPCAT-5) (Lyso-PC acyltransferase 5) (EC 2.3.1.23) (Non-selenium glutathione peroxidase) (NSGPx)	Prdx6 Aop2 Ltw4 Prdx5	Only in WT-iAstro cells
CSTN1_MOUSE	Q9EPL2	Calsyntenin-1 (Alcadein-alpha) (Alc-alpha) [Cleaved into: Soluble Alc-alpha (SAlc-alpha); CTF1-alpha (C-terminal fragment 1-alpha)]	Clstn1 Cs1 Cstn1 Kiaa0911	Only in WT-iAstro cells

H2B3B_MOUSE	Q8CGP0	Histone H2B type 3-B (H2B.U histone 1)	H2bu1 H2bu1-ps Hist3h2bb Hist3h2bb-ps	Only in WT-iAstro cells
TBA4A_MOUSE	P68368	Tubulin alpha-4A chain (Alpha-tubulin 4) (Alpha-tubulin isotype M-alpha-4) (Tubulin alpha-4 chain)	Tuba4a Tuba4	Only in WT-iAstro cells
CNTRL_MOUSE	A2AL36	Centriolin (Centrosomal protein 1) (Centrosomal protein of 110 kDa) (Cep110)	Cntrl Cep1 Cep110	Only in WT-iAstro cells
1433E_MOUSE	P62259	14-3-3 protein epsilon (14-3-3E)	Ywhae	Only in WT-iAstro cells
CO5A1_MOUSE	O88207	Collagen alpha-1(V) chain	Col5a1	Only in WT-iAstro cells
TSSK6_MOUSE	Q925K9	Testis-specific serine/threonine-protein kinase 6 (TSK-6) (TSSK-6) (Testis-specific kinase 6) (EC 2.7.11.1) (Serine/threonine-protein kinase SSTK) (Small serine/threonine kinase)	Tssk6 Sstk	Only in WT-iAstro cells
PROF1_MOUSE	P62962	Profilin-1 (Profilin I)	Pfn1	Only in WT-iAstro cells
CSF1_MOUSE	P07141	Macrophage colony-stimulating factor 1 (CSF-1) (MCSF) [Cleaved into: Processed macrophage colony-stimulating factor 1]	Csf1 Csfm	Only in WT-iAstro cells
HSP7C_MOUSE	P63017	Heat shock cognate 71 kDa protein (EC 3.6.4.10) (Heat shock 70 kDa protein 8)	Hspa8 Hsc70 Hsc73	Only in WT-iAstro cells
HTRA1_MOUSE	Q9R118	Serine protease HTRA1 (EC 3.4.21.-) (High-temperature requirement A serine peptidase 1) (Serine protease 11)	Htra1 Htra Prss11	Only in WT-iAstro cells

MIF_MOUSE	P34884	Macrophage migration inhibitory factor (MIF) (EC 5.3.2.1) (Delayed early response protein 6) (DER6) (Glycosylation-inhibiting factor) (GIF) (L-dopachrome isomerase) (L-dopachrome tautomerase) (EC 5.3.3.12) (Phenylpyruvate tautomerase)	Mif	Only in WT-iAstro cells
S10A4_MOUSE	P07091	Protein S100-A4 (Metastasin) (Metastatic cell protein) (PEL98) (Placental calcium-binding protein) (Protein 18A2) (Protein Mts1) (S100 calcium-binding protein A4)	S100a4 Capl Mts1	Only in WT-iAstro cells
TPM3_MOUSE	P21107	Tropomyosin alpha-3 chain (Gamma-tropomyosin) (Tropomyosin-3)	Tpm3 Tpm-5 Tpm5	Only in WT-iAstro cells
MMP3_MOUSE	P28862	Stromelysin-1 (SL-1) (EC 3.4.24.17) (EMS-2) (Matrix metalloproteinase-3) (MMP-3) (Transin-1)	Mmp3	Only in WT-iAstro cells
HBA_MOUSE	P01942	Hemoglobin subunit alpha (Alpha-globin) (Hemoglobin alpha chain)	Hba Hba-a1	Only in WT-iAstro cells
PGS1_MOUSE	P28653	Biglycan (Bone/cartilage proteoglycan I) (PG-S1)	Bgn	Only in WT-iAstro cells
MOES_MOUSE	P26041	Moesin (Membrane-organizing extension spike protein)	Msn	Only in WT-iAstro cells
CAV2_MOUSE	Q9WVC3	Caveolin-2	Cav2	Only in WT-iAstro cells
CO6A1_MOUSE	Q04857	Collagen alpha-1(VI) chain	Col6a1	Only in WT-iAstro cells
ENOA_MOUSE	P17182	Alpha-enolase (EC 4.2.1.11) (2-phospho-D-glycerate hydro-lyase) (Enolase 1) (Non-neural enolase) (NNE)	Eno1 Eno-1	Only in WT-iAstro cells
ECM1_MOUSE	Q61508	Extracellular matrix protein 1 (Secretory component p85)	Ecm1	Only in WT-iAstro cells

PGAM1_MOUSE	Q9DBJ1	Phosphoglycerate mutase 1 (EC 5.4.2.11) (EC 5.4.2.4) (BPG-dependent PGAM 1) (Phosphoglycerate mutase isozyme B) (PGAM-B)	Pgam1	Only in WT-iAstro cells
PDC6I_MOUSE	Q9WU78	Programmed cell death 6-interacting protein (ALG-2-interacting protein 1) (ALG-2-interacting protein X) (E2F1-inducible protein) (Eig2)	Pdcd6ip Aip1 Alix	Only in WT-iAstro cells
PPARD_MOUSE	P35396	Peroxisome proliferator-activated receptor delta (PPAR-delta) (Nuclear hormone receptor 1) (NUC1) (Nuclear receptor subfamily 1 group C member 2) (Peroxisome proliferator-activated receptor beta) (PPAR-beta)	Ppard Nr1c2 Pparb	Only in WT-iAstro cells
K2C8_MOUSE	P11679	Keratin, type II cytoskeletal 8 (Cytokeratin endo A) (Cytokeratin-8) (CK-8) (Keratin-8) (K8) (Type-II keratin Kb8)	Krt8 Krt2-8	Only in WT-iAstro cells
LDHA_MOUSE	P06151	L-lactate dehydrogenase A chain (LDH-A) (EC 1.1.1.27) (LDH muscle subunit) (LDH-M)	Ldha Ldh-1 Ldh1	Only in WT-iAstro cells
PCOC1_MOUSE	Q61398	Procollagen C-endopeptidase enhancer 1 (P14) (Procollagen COOH-terminal proteinase enhancer 1) (PCPE-1) (Procollagen C-proteinase enhancer 1) (Type 1 procollagen C-proteinase enhancer protein) (Type I procollagen COOH-terminal proteinase enhancer)	Pcolce Pcpe1	Only in WT-iAstro cells
CATB_MOUSE	P10605	Cathepsin B (EC 3.4.22.1) (Cathepsin B1) [Cleaved into: Cathepsin B light chain; Cathepsin B heavy chain]	Ctsb	Only in WT-iAstro cells

FBLN5_MOUSE	Q9WVH9	Fibulin-5 (FIBL-5) (Developmental arteries and neural crest EGF-like protein) (Dance)	Fbln5 Dance	Only in WT-iAstro cells
1433Z_MOUSE	P63101	14-3-3 protein zeta/delta (Protein kinase C inhibitor protein 1) (KCIP-1) (SEZ-2)	Ywhaz	Only in WT-iAstro cells
VINC_MOUSE	Q64727	Vinculin (Metavinculin)	Vcl	Only in WT-iAstro cells
SEM5B_MOUSE	Q60519	Semaphorin-5B (Semaphorin-G) (Sema G)	Sema5b Kiaa1445 Semag SemG	Only in WT-iAstro cells
EF1A1_MOUSE	P10126	Elongation factor 1-alpha 1 (EF-1-alpha-1) (Elongation factor Tu) (EF-Tu) (Eukaryotic elongation factor 1 A-1) (eEF1A-1)	Eef1a1 Eef1a	Only in WT-iAstro cells
TTC25_MOUSE	Q9D4B2	Outer dynein arm-docking complex subunit 4 (Tetratricopeptide repeat protein 25) (TPR repeat protein 25)	Odad4 Ttc25	Only in WT-iAstro cells
LDHB_MOUSE	P16125	L-lactate dehydrogenase B chain (LDH-B) (EC 1.1.1.27) (LDH heart subunit) (LDH-H)	Ldhb Ldh-2 Ldh2	Only in WT-iAstro cells
PCSK9_MOUSE	Q80W65	Proprotein convertase subtilisin/kexin type 9 (EC 3.4.21.-) (Neural apoptosis-regulated convertase 1) (NARC-1) (Proprotein convertase 9) (PC9) (Subtilisin/kexin-like protease PC9)	Pcsk9 Narc1	Only in WT-iAstro cells
RLA0_MOUSE	P14869	60S acidic ribosomal protein P0 (60S ribosomal protein L10E)	Rplp0 Arbp	Only in WT-iAstro cells
PRAL7_MOUSE	Q810Y8	Preferentially expressed antigen in melanoma-like protein 7 (Prame-like 7)	Pramel7	Only in WT-iAstro cells
CO1A1_MOUSE	P11087	Collagen alpha-1(I) chain (Alpha-1 type I collagen)	Col1a1 Cola1	Only in WT-iAstro cells
EF2_MOUSE	P58252	Elongation factor 2 (EF-2)	Eef2	Only in WT-iAstro cells

ABCA1_MOUSE	P41233	Phospholipid-transporting ATPase ABCA1 (EC 7.6.2.1) (ATP-binding cassette sub-family A member 1) (ATP-binding cassette transporter 1) (ABC-1) (ATP-binding cassette 1)	Abca1 Abc1	Only in WT-iAstro cells
FLNA_MOUSE	Q8BTM8	Filamin-A (FLN-A) (Actin-binding protein 280) (ABP-280) (Alpha-filamin) (Endothelial actin-binding protein) (Filamin-1) (Non-muscle filamin)	Flna Fln Fln1	Only in WT-iAstro cells
LIFR_MOUSE	P42703	Leukemia inhibitory factor receptor (LIF receptor) (LIF-R) (D-factor/LIF receptor) (CD antigen CD118)	Lifr	Only in WT-iAstro cells
PRDX1_MOUSE	P35700	Peroxiredoxin-1 (EC 1.11.1.24) (Macrophage 23 kDa stress protein) (Osteoblast-specific factor 3) (OSF-3) (Thioredoxin peroxidase 2) (Thioredoxin-dependent peroxide reductase 2) (Thioredoxin-dependent peroxiredoxin 1)	Prdx1 Msp23 Paga Tdpx2	Only in WT-iAstro cells
LYOX_MOUSE	P28301	Protein-lysine 6-oxidase (EC 1.4.3.13) (Lysyl oxidase) (Ras excision protein) [Cleaved into: Protein-lysine 6-oxidase, long form; Protein-lysine 6-oxidase, short form]	Lox Rrg	Only in WT-iAstro cells
1433G_MOUSE	P61982	14-3-3 protein gamma [Cleaved into: 14-3-3 protein gamma, N-terminally processed]	Ywhag	Only in WT-iAstro cells
ITIH1_MOUSE	Q61702	Inter-alpha-trypsin inhibitor heavy chain H1 (ITI heavy chain H1) (ITI-HC1) (Inter-alpha-inhibitor heavy chain 1)	Itih1	Only in WT-iAstro cells

G3P_MOUSE	P16858	Glyceraldehyde-3-phosphate dehydrogenase (GAPDH) (EC 1.2.1.12) (Peptidyl-cysteine S-nitrosylase GAPDH) (EC 2.6.99.-)	Gapdh Gapd	Only in WT-iAstro cells
TRFL_MOUSE	P08071	Lactotransferrin (Lactoferrin) (EC 3.4.21.-)	Ltf	Only in 3Tg-iAstro cells
PR7C1_MOUSE	Q9CRB5	Prolactin-7C1 (Placental prolactin-like protein O) (PLP-O) (PRL-like protein O)	Prl7c1 Prlpo	Only in 3Tg-iAstro cells
ARF5_MOUSE	P84084	ADP-ribosylation factor 5	Arf5	Only in 3Tg-iAstro cells
COF1_MOUSE	P18760	Cofilin-1 (Cofilin, non-muscle isoform)	Cfl1	Only in 3Tg-iAstro cells
TREA_MOUSE	Q9JLT2	Trehalase (EC 3.2.1.28) (Alpha,alpha-trehalase) (Alpha,alpha-trehalose glucohydrolase)	Treh	Only in 3Tg-iAstro cells
FABPH_MOUSE	P11404	Fatty acid-binding protein, heart (Fatty acid-binding protein 3) (Heart-type fatty acid-binding protein) (H-FABP) (Mammary-derived growth inhibitor) (MDGI)	Fabp3 Fabph1	Only in 3Tg-iAstro cells
TRAP1_MOUSE	Q9CQN1	Heat shock protein 75 kDa, mitochondrial (HSP 75) (TNFR-associated protein 1) (Tumor necrosis factor type 1 receptor-associated protein) (TRAP-1)	Trap1 Hsp75	Only in 3Tg-iAstro cells
TPM2_MOUSE	P58774	Tropomyosin beta chain (Beta-tropomyosin) (Tropomyosin-2)	Tpm2 Tpm-2	Only in 3Tg-iAstro cells
STAG1_MOUSE	Q9D3E6	Cohesin subunit SA-1 (SCC3 homolog 1) (Stromal antigen 1)	Stag1 Sa1	Only in 3Tg-iAstro cells
K1C13_MOUSE	P08730	Keratin, type I cytoskeletal 13 (47 kDa cytokeratin) (Cytokeratin-13) (CK-13) (Keratin-13) (K13)	Krt13 Krt1-13	Only in 3Tg-iAstro cells

CFAB_MOUSE	P04186	Complement factor B (EC 3.4.21.47) (C3/C5 convertase) [Cleaved into: Complement factor B Ba fragment; Complement factor B Bb fragment]	Cfb Bf H2-Bf	Only in 3Tg-iAstro cells
K1C15_MOUSE	Q61414	Keratin, type I cytoskeletal 15 (Cytokeratin-15) (CK-15) (Keratin-15) (K15)	Krt15 Krt1-15	Only in 3Tg-iAstro cells
PLXD1_MOUSE	Q3UH93	Plexin-D1	Plxnd1	Only in 3Tg-iAstro cells
COCA1_MOUSE	Q60847	Collagen alpha-1(XII) chain	Col12a1	Only in 3Tg-iAstro cells
PAI1_MOUSE	P22777	Plasminogen activator inhibitor 1 (PAI) (PAI-1) (Endothelial plasminogen activator inhibitor) (Serpin E1)	Serpine1 Mr1 Pai1 Planh1	Only in 3Tg-iAstro cells
B4GT1_MOUSE	P15535	Beta-1,4-galactosyltransferase 1 (Beta-1,4-GalTase 1) (Beta4Gal-T1) (b4Gal-T1) (EC 2.4.1.-) (Beta-N-acetylglucosaminyl-glycolipid beta-1,4-galactosyltransferase) (Beta-N-acetylglucosaminylglycopeptide beta-1,4-galactosyltransferase) (EC 2.4.1.38) (Lactose synthase A protein) (EC 2.4.1.22) (N-acetyllactosamine synthase) (EC 2.4.1.90) (Nal synthase) (Neolactotriaosylceramide beta-1,4-galactosyltransferase) (EC 2.4.1.275) (UDP-Gal:beta-GlcNAc beta-1,4-galactosyltransferase 1) (UDP-galactose:beta-N-acetylglucosamine beta-1,4-galactosyltransferase 1) [Cleaved into: Processed beta-1,4-galactosyltransferase 1]	B4galt1 Ggtb Ggtb2	Only in 3Tg-iAstro cells
NEST_MOUSE	Q6P5H2	Nestin	Nes	Only in 3Tg-iAstro cells

FABP4_MOUSE	P04117	Fatty acid-binding protein, adipocyte (3T3-L1 lipid-binding protein) (Adipocyte lipid-binding protein) (ALBP) (Adipocyte-type fatty acid-binding protein) (A-FABP) (AFABP) (Fatty acid-binding protein 4) (Myelin P2 protein homolog) (P15) (P2 adipocyte protein) (Protein 422)	Fabp4 Ap2	Only in 3Tg-iAstro cells
ACTN4_MOUSE	P57780	Alpha-actinin-4 (Non-muscle alpha-actinin 4)	Actn4	Only in 3Tg-iAstro cells
PON1_MOUSE	P52430	Serum paraoxonase/arylesterase 1 (PON 1) (EC 3.1.1.2) (EC 3.1.1.81) (EC 3.1.8.1) (Aromatic esterase 1) (A-esterase 1) (Serum aryldialkylphosphatase 1)	Pon1 Pon	Only in 3Tg-iAstro cells
LSM4_MOUSE	Q9QXA5	U6 snRNA-associated Sm-like protein LSm4	Lsm4	Only in 3Tg-iAstro cells
XDH_MOUSE	Q00519	Xanthine dehydrogenase/oxidase [Includes: Xanthine dehydrogenase (XD) (EC 1.17.1.4); Xanthine oxidase (XO) (EC 1.17.3.2) (Xanthine oxidoreductase) (XOR)]	Xdh	Only in 3Tg-iAstro cells
FA8_MOUSE	Q06194	Coagulation factor VIII (Procoagulant component)	F8 Cf8 F8c	Only in 3Tg-iAstro cells
ACTBL_MOUSE	Q8BFZ3	Beta-actin-like protein 2 (Kappa-actin)	Actbl2	Only in 3Tg-iAstro cells
HBB1_MOUSE	P02088	Hemoglobin subunit beta-1 (Beta-1-globin) (Hemoglobin beta-1 chain) (Hemoglobin beta-major chain)	Hbb-b1	Only in 3Tg-iAstro cells
NID2_MOUSE	O88322	Nidogen-2 (NID-2) (Entactin-2)	Nid2	Only in 3Tg-iAstro cells
K1C16_MOUSE	Q9Z2K1	Keratin, type I cytoskeletal 16 (Cytokeratin-16) (CK-16) (Keratin-16) (K16)	Krt16 Krt1-16	Only in 3Tg-iAstro cells
SPA3M_MOUSE	Q03734	Serine protease inhibitor A3M (Serpin A3M)	Serpina3m	Only in 3Tg-iAstro cells

ACTA_MOUSE	P62737	Actin, aortic smooth muscle (Alpha-actin-2) [Cleaved into: Actin, aortic smooth muscle, intermediate form]	Acta2 Actsa Actvs	Only in 3Tg-iAstro cells
