# Supplementary material for: Protein synthesis inhibition and loss of homeostatic functions in astrocytes from an Alzheimer’s disease mouse model: a role for ER-mitochondria interaction
Source: Cell Death Dis. 2022 Oct 18;13(10):878. doi: 10.1038/s41419-022-05324-4 (PMC9579125; doi:10.1038/s41419-022-05324-4)
Supplement: Supplementary file 4 — Supplemental table 2 [file 41419_2022_5324_MOESM4_ESM.pdf]

Supplemental Table 2.

Shotgun mass spectrometry proteomics of ACM from WT-iAstro and 3Tg-iAstro cells

Differentially expressed proteins

N = 3 independent cultures for each genotype

Uniprot_ID	Description	Gene	p-value	Fold Change
FABPH_MOUSE	Fatty acid-binding protein, heart	Fabp3	0.044	4.74
SPRC_MOUSE	SPARC	Sparc	0.027	-1.59
HS90B_MOUSE	Heat shock protein HSP 90-beta	Hsp90ab1	0.037	-2.14
HSP7C_MOUSE	Heat shock cognate 71 kDa protein	Hspa8	0.041	-2.33
TBA1B_MOUSE	Tubulin alpha-1B chain	Tuba1b	0.021	-3.95
