# Supplementary material for: Protein synthesis inhibition and loss of homeostatic functions in astrocytes from an Alzheimer’s disease mouse model: a role for ER-mitochondria interaction
Source: Cell Death Dis. 2022 Oct 18;13(10):878. doi: 10.1038/s41419-022-05324-4 (PMC9579125; doi:10.1038/s41419-022-05324-4)
Supplement: Supplementary file 5 — Supplemental table3 [file 41419_2022_5324_MOESM5_ESM.pdf]

Supplemental Table 3.

Shotgun mass spectrometry proteomics of ACM from WT-iAstro and 10nm-EML-expressing WT-iAstro cells

Identified proteins

N = 3 independent cultures for each condition

Uniptor_ID	Uniprot_KB	Description	Identified in:
KPYM_MOUSE	P52480	Pyruvate kinase PKM	WT-iAstro and 10nm-EML-expressing WT-iAstro cells
PPIA_MOUSE	P17742	Peptidyl-prolyl cis-trans isomerase A	WT-iAstro and 10nm-EML-expressing WT-iAstro cells
HS90B_MOUSE	P11499	Heat shock protein HSP 90-beta	WT-iAstro and 10nm-EML-expressing WT-iAstro cells
NRK_MOUSE	Q9R0G8	Nik-related protein kinase	WT-iAstro and 10nm-EML-expressing WT-iAstro cells
ALBU_MOUSE	P07724	Albumin	WT-iAstro and 10nm-EML-expressing WT-iAstro cells
K2C75_MOUSE	Q8BGZ7	Keratin, type II cytoskeletal 75	WT-iAstro and 10nm-EML-expressing WT-iAstro cells
A2MG_MOUSE	Q6GQT1	Alpha-2-macroglobulin-P	WT-iAstro and 10nm-EML-expressing WT-iAstro cells
ITIH3_MOUSE	Q61704	Inter-alpha-trypsin inhibitor heavy chain H3	WT-iAstro and 10nm-EML-expressing WT-iAstro cells
SPRC_MOUSE	P07214	SPARC	WT-iAstro and 10nm-EML-expressing WT-iAstro cells
FINC_MOUSE	P11276	Fibronectin	WT-iAstro and 10nm-EML-expressing WT-iAstro cells
FBLN1_MOUSE	Q08879	Fibulin-1	WT-iAstro and 10nm-EML-expressing WT-iAstro cells
ANT3_MOUSE	P32261	Antithrombin-III	WT-iAstro and 10nm-EML-expressing WT-iAstro cells
K2C79_MOUSE	Q8VED5	Keratin, type II cytoskeletal 79	WT-iAstro and 10nm-EML-expressing WT-iAstro cells
K1C10_MOUSE	P02535	Keratin, type I cytoskeletal 10	WT-iAstro and 10nm-EML-expressing WT-iAstro cells
CO3_MOUSE	P01027	Complement C3	WT-iAstro and 10nm-EML-expressing WT-iAstro cells
TSP1_MOUSE	P35441	Thrombospondin-1	WT-iAstro and 10nm-EML-expressing WT-iAstro cells
PZP_MOUSE	Q61838	Pregnancy zone protein	WT-iAstro and 10nm-EML-expressing WT-iAstro cells
ACTB_MOUSE	P60710	Actin, cytoplasmic 1	WT-iAstro and 10nm-EML-expressing WT-iAstro cells
ITIH2_MOUSE	Q61703	Inter-alpha-trypsin inhibitor heavy chain H2	WT-iAstro and 10nm-EML-expressing WT-iAstro cells

K2C1_MOUSE	P04104	Keratin, type II cytoskeletal 1	WT-iAstro and 10nm-EML-expressing WT-iAstro cells
PEDF_MOUSE	P97298	Pigment epithelium-derived factor	WT-iAstro and 10nm-EML-expressing WT-iAstro cells
RS27A_MOUSE	P62983	Ubiquitin-40S ribosomal protein S27a	WT-iAstro and 10nm-EML-expressing WT-iAstro cells
CO1A2_MOUSE	Q01149	Collagen alpha-2(I) chain	WT-iAstro and 10nm-EML-expressing WT-iAstro cells
B2MG_MOUSE	P01887	Beta-2-microglobulin	WT-iAstro and 10nm-EML-expressing WT-iAstro cells
ACTN1_MOUSE	Q7TPR4	Alpha-actinin-1	WT-iAstro and 10nm-EML-expressing WT-iAstro cells
PROF1_MOUSE	P62962	Profilin-1	WT-iAstro and 10nm-EML-expressing WT-iAstro cells
HSP7C_MOUSE	P63017	Heat shock cognate 71 kDa protein	WT-iAstro and 10nm-EML-expressing WT-iAstro cells
MIF_MOUSE	P34884	Macrophage migration inhibitory factor	WT-iAstro and 10nm-EML-expressing WT-iAstro cells
TPM3_MOUSE	P21107	Tropomyosin alpha-3 chain	WT-iAstro and 10nm-EML-expressing WT-iAstro cells
PGS1_MOUSE	P28653	Biglycan	WT-iAstro and 10nm-EML-expressing WT-iAstro cells
ENOA_MOUSE	P17182	Alpha-enolase	WT-iAstro and 10nm-EML-expressing WT-iAstro cells
K2C8_MOUSE	P11679	Keratin, type II cytoskeletal 8	WT-iAstro and 10nm-EML-expressing WT-iAstro cells
LDHA_MOUSE	P06151	L-lactate dehydrogenase A chain	WT-iAstro and 10nm-EML-expressing WT-iAstro cells
CO1A1_MOUSE	P11087	Collagen alpha-1(I) chain	WT-iAstro and 10nm-EML-expressing WT-iAstro cells
SYCP1_MOUSE	Q62209	Synaptonemal complex protein 1	Only WT-iAstro cells
FSTL1_MOUSE	Q62356	Follistatin-related protein 1	Only WT-iAstro cells
VIME_MOUSE	P20152	Vimentin	Only WT-iAstro cells
OSTP_MOUSE	P10923	Osteopontin	Only WT-iAstro cells
AFAM_MOUSE	O89020	Afamin (Alpha-albumin) (Alpha-Alb)	Only WT-iAstro cells
CATD_MOUSE	P18242	Cathepsin D	Only WT-iAstro cells
NRAP_MOUSE	Q80XB4	Nebulin-related-anchoring protein	Only WT-iAstro cells
K22O_MOUSE	Q3UV17	Keratin, type II cytoskeletal 2 oral	Only WT-iAstro cells
VNN3_MOUSE	Q9QZ25	Vascular non-inflammatory molecule 3	Only WT-iAstro cells
CBPE_MOUSE	Q00493	Carboxypeptidase E	Only WT-iAstro cells
FA92A_MOUSE	Q8BP22	Protein FAM92A	Only WT-iAstro cells

RC3H2_MOUSE	P0C090	Roquin-2	Only WT-iAstro cells
TBA1B_MOUSE	P05213	Tubulin alpha-1B chain	Only WT-iAstro cells
TTHY_MOUSE	P07309	Transthyretin	Only WT-iAstro cells
ITIH4_MOUSE	A6X935	Inter alpha-trypsin inhibitor, heavy chain 4	Only WT-iAstro cells
SAP_MOUSE	Q61207	Prosaposin	Only WT-iAstro cells
NUCB1_MOUSE	Q02819	Nucleobindin-1	Only WT-iAstro cells
TRFE_MOUSE	Q921I1	Serotransferrin	Only WT-iAstro cells
DYST_MOUSE	Q91ZU6	Dystonin	Only WT-iAstro cells
IBP2_MOUSE	P47877	Insulin-like growth factor-binding protein 2	Only WT-iAstro cells
ARAP3_MOUSE	Q8R5G7	Arf-GAP with Rho-GAP domain, ANK repeat and PH domain-containing protein 3	Only WT-iAstro cells
FETUA_MOUSE	P29699	Alpha-2-HS-glycoprotein	Only WT-iAstro cells
CO4B_MOUSE	P01029	Complement C4-B	Only WT-iAstro cells
LUM_MOUSE	P51885	Lumican	Only WT-iAstro cells
HBB2_MOUSE	P02089	Hemoglobin subunit beta-2	Only WT-iAstro cells
TPM4_MOUSE	Q6IRU2	Tropomyosin alpha-4 chain	Only WT-iAstro cells
TENA_MOUSE	Q80YX1	Tenascin	Only WT-iAstro cells
PLMN_MOUSE	P20918	Plasminogen	Only WT-iAstro cells
K2C1B_MOUSE	Q6IFZ6	Keratin, type II cytoskeletal 1b	Only WT-iAstro cells
APOA1_MOUSE	Q00623	Apolipoprotein A-I	Only WT-iAstro cells
ACTG_MOUSE	P63260	Actin, cytoplasmic 2 (Gamma-actin)	Only WT-iAstro cells
MUG1_MOUSE	P28665	Murinoglobulin-1	Only WT-iAstro cells
KNG1_MOUSE	O08677	Kininogen-1	Only WT-iAstro cells
ACTC_MOUSE	P68033	Actin, alpha cardiac muscle 1	Only WT-iAstro cells
DCTP1_MOUSE	Q9QY93	dCTP pyrophosphatase 1	Only WT-iAstro cells
HEXA_MOUSE	P29416	Beta-hexosaminidase subunit alpha	Only WT-iAstro cells
1433B_MOUSE	Q9CQV8	14-3-3 protein beta/alpha	Only WT-iAstro cells
LRP5_MOUSE	Q91VN0	Low-density lipoprotein receptor-related protein 5 (LRP-5)	Only WT-iAstro cells

NPC2_MOUSE	Q9Z0J0	NPC intracellular cholesterol transporter 2	Only WT-iAstro cells
RL40_MOUSE	P62984	Ubiquitin-60S ribosomal protein L40	Only WT-iAstro cells
HS90A_MOUSE	P07901	Heat shock protein HSP 90-alpha	Only WT-iAstro cells
H3C_MOUSE	P02301	Histone H3.3C	Only WT-iAstro cells
NDKB_MOUSE	Q01768	Nucleoside diphosphate kinase B	Only WT-iAstro cells
TPM1_MOUSE	P58771	Tropomyosin alpha-1 chain	Only WT-iAstro cells
CRLF1_MOUSE	Q9JM58	Cytokine receptor-like factor 1	Only WT-iAstro cells
PRDX6_MOUSE	O08709	Peroxiredoxin-6	Only WT-iAstro cells
CSTN1_MOUSE	Q9EPL2	Calsyntenin-1	Only WT-iAstro cells
H2B3B_MOUSE	Q8CGP0	Histone H2B type 3-B	Only WT-iAstro cells
TBA4A_MOUSE	P68368	Tubulin alpha-4A chain	Only WT-iAstro cells
CNTRL_MOUSE	A2AL36	Centriolin	Only WT-iAstro cells
1433E_MOUSE	P62259	14-3-3 protein epsilon	Only WT-iAstro cells
CO5A1_MOUSE	O88207	Collagen alpha-1(V) chain	Only WT-iAstro cells
TSSK6_MOUSE	Q925K9	Testis-specific serine/threonine-protein kinase 6	Only WT-iAstro cells
CSF1_MOUSE	P07141	Macrophage colony-stimulating factor 1	Only WT-iAstro cells
HTRA1_MOUSE	Q9R118	Serine protease HTRA1	Only WT-iAstro cells
S10A4_MOUSE	P07091	Protein S100-A4	Only WT-iAstro cells
MMP3_MOUSE	P28862	Stromelysin-1	Only WT-iAstro cells
HBA_MOUSE	P01942	Hemoglobin subunit alpha	Only WT-iAstro cells
MOES_MOUSE	P26041	Moesin	Only WT-iAstro cells
CAV2_MOUSE	Q9WVC3	Caveolin-2	Only WT-iAstro cells
CO6A1_MOUSE	Q04857	Collagen alpha-1(VI) chain	Only WT-iAstro cells
ECM1_MOUSE	Q61508	Extracellular matrix protein 1	Only WT-iAstro cells
PGAM1_MOUSE	Q9DBJ1	Phosphoglycerate mutase 1	Only WT-iAstro cells
PDC6I_MOUSE	Q9WU78	Programmed cell death 6-interacting protein	Only WT-iAstro cells
PPARD_MOUSE	P35396	Peroxisome proliferator-activated receptor delta (PPAR-delta)	Only WT-iAstro cells

PCOC1_MOUSE	Q61398	Procollagen C-endopeptidase enhancer 1	Only WT-iAstro cells
CATB_MOUSE	P10605	Cathepsin B	Only WT-iAstro cells
FBLN5_MOUSE	Q9WVH9	Fibulin-5	Only WT-iAstro cells
1433Z_MOUSE	P63101	14-3-3 protein zeta/delta	Only WT-iAstro cells
VINC_MOUSE	Q64727	Vinculin	Only WT-iAstro cells
SEM5B_MOUSE	Q60519	Semaphorin-5B	Only WT-iAstro cells
EF1A1_MOUSE	P10126	Elongation factor 1-alpha 1	Only WT-iAstro cells
TTC25_MOUSE	Q9D4B2	Outer dynein arm-docking complex subunit 4	Only WT-iAstro cells
LDHB_MOUSE	P16125	L-lactate dehydrogenase B chain	Only WT-iAstro cells
PCSK9_MOUSE	Q80W65	Proprotein convertase subtilisin/kexin type 9	Only WT-iAstro cells
RLA0_MOUSE	P14869	60S acidic ribosomal protein P0	Only WT-iAstro cells
PRAL7_MOUSE	Q810Y8	Preferentially expressed antigen in melanoma-like protein 7	Only WT-iAstro cells
EF2_MOUSE	P58252	Elongation factor 2	Only WT-iAstro cells
ABCA1_MOUSE	P41233	Phospholipid-transporting ATPase ABCA1	Only WT-iAstro cells
FLNA_MOUSE	Q8BTM8	Filamin-A (FLN-A)	Only WT-iAstro cells
LIFR_MOUSE	P42703	Leukemia inhibitory factor receptor	Only WT-iAstro cells
PRDX1_MOUSE	P35700	Peroxiredoxin-1	Only WT-iAstro cells
LYOX_MOUSE	P28301	Protein-lysine 6-oxidase	Only WT-iAstro cells
1433G_MOUSE	P61982	14-3-3 protein gamma	Only WT-iAstro cells
ITIH1_MOUSE	Q61702	Inter-alpha-trypsin inhibitor heavy chain H1	Only WT-iAstro cells
G3P_MOUSE	P16858	Glyceraldehyde-3-phosphate dehydrogenase (GAPDH)	Only WT-iAstro cells
S10A6_MOUSE	P14069	Protein S100-A6	Only in 10nm-EML-expressing WT-iAstro cells
TAGL2_MOUSE	Q9WVA4	Transgelin-2	Only in 10nm-EML-expressing WT-iAstro cells
SODC_MOUSE	P08228	Superoxide dismutase [Cu-Zn]	Only in 10nm-EML-expressing WT-iAstro cells
ABHD8_MOUSE	Q8R0P8	Protein ABHD8	Only in 10nm-EML-expressing WT-iAstro cells

ILRL1_MOUSE	P14719	Interleukin-1 receptor-like 1	Only in 10nm-EML-expressing WT-iAstro cells
CS047_MOUSE	Q8R3Y5	Uncharacterized protein C19orf47 homolog	Only in 10nm-EML-expressing WT-iAstro cells
