# Supplementary material for: Protein synthesis inhibition and loss of homeostatic functions in astrocytes from an Alzheimer’s disease mouse model: a role for ER-mitochondria interaction
Source: Cell Death Dis. 2022 Oct 18;13(10):878. doi: 10.1038/s41419-022-05324-4 (PMC9579125; doi:10.1038/s41419-022-05324-4)
Supplement: Supplementary file 6 — Supplemental table4 [file 41419_2022_5324_MOESM6_ESM.pdf]

Supplemental Table 4.

Shotgun mass spectrometry proteomics of ACM from 3Tg-iAstro and 4-PBA-treated 3Tg-iAstro cells

Identified proteins

N = 3 independent cultures for each genotype

Uniptor_ID	Uniprot_KB	Description	Identified in:
SYCP1_MOUSE	Q62209	Synaptonemal complex protein 1	3Tg-iAstro and 4-PBA-treated 3Tg-iAstro cells
KPYM_MOUSE	P52480-2	Isoform M1 of Pyruvate kinase PKM	3Tg-iAstro and 4-PBA-treated 3Tg-iAstro cells
PPIA_MOUSE	P17742	Peptidyl-prolyl cis-trans isomerase A	3Tg-iAstro and 4-PBA-treated 3Tg-iAstro cells
FSTL1_MOUSE	Q62356	Follistatin-related protein 1	3Tg-iAstro and 4-PBA-treated 3Tg-iAstro cells
VIME_MOUSE	P20152	Vimentin	3Tg-iAstro and 4-PBA-treated 3Tg-iAstro cells
HS90B_MOUSE	P11499	Heat shock protein HSP 90-beta	3Tg-iAstro and 4-PBA-treated 3Tg-iAstro cells
ALBU_MOUSE	P07724	Serum albumin	3Tg-iAstro and 4-PBA-treated 3Tg-iAstro cells
OSTP_MOUSE	P10923	Osteopontin	3Tg-iAstro and 4-PBA-treated 3Tg-iAstro cells
AFAM_MOUSE	O89020-3	Isoform 3 of Afamin	3Tg-iAstro and 4-PBA-treated 3Tg-iAstro cells
CATD_MOUSE	P18242	Cathepsin D	3Tg-iAstro and 4-PBA-treated 3Tg-iAstro cells
A2MG_MOUSE	Q6GQT1	Alpha-2-macroglobulin-P	3Tg-iAstro and 4-PBA-treated 3Tg-iAstro cells
K22O_MOUSE	Q3UV17	Keratin, type II cytoskeletal 2 oral	3Tg-iAstro and 4-PBA-treated 3Tg-iAstro cells
ITIH3_MOUSE	Q61704	Inter-alpha-trypsin inhibitor heavy chain H3	3Tg-iAstro and 4-PBA-treated 3Tg-iAstro cells
SPRC_MOUSE	P07214	SPARC	3Tg-iAstro and 4-PBA-treated 3Tg-iAstro cells
CBPE_MOUSE	Q00493	Carboxypeptidase E	3Tg-iAstro and 4-PBA-treated 3Tg-iAstro cells
FINC_MOUSE	P11276	Fibronectin	3Tg-iAstro and 4-PBA-treated 3Tg-iAstro cells
TBA1B_MOUSE	P05213	Tubulin alpha-1B chain	3Tg-iAstro and 4-PBA-treated 3Tg-iAstro cells
FBLN1_MOUSE	Q08879	Fibulin-1	3Tg-iAstro and 4-PBA-treated 3Tg-iAstro cells
SAP_MOUSE	Q61207	Prosaposin	3Tg-iAstro and 4-PBA-treated 3Tg-iAstro cells
ANT3_MOUSE	P32261	Antithrombin-III	3Tg-iAstro and 4-PBA-treated 3Tg-iAstro cells
K2C79_MOUSE	Q8VED5	Keratin, type II cytoskeletal 79	3Tg-iAstro and 4-PBA-treated 3Tg-iAstro cells

K1C10_MOUSE	P02535-2	Isoform 2 of Keratin, type I cytoskeletal 10	3Tg-iAstro and 4-PBA-treated 3Tg-iAstro cells
DYST_MOUSE	Q91ZU6-3	Isoform 3 of Dystonin	3Tg-iAstro and 4-PBA-treated 3Tg-iAstro cells
CO3_MOUSE	P01027	Complement C3	3Tg-iAstro and 4-PBA-treated 3Tg-iAstro cells
IBP2_MOUSE	P47877	Insulin-like growth factor-binding protein 2	3Tg-iAstro and 4-PBA-treated 3Tg-iAstro cells
FETUA_MOUSE	P29699	Alpha-2-HS-glycoprotein	3Tg-iAstro and 4-PBA-treated 3Tg-iAstro cells
CO4B_MOUSE	P01029	Complement C4-B	3Tg-iAstro and 4-PBA-treated 3Tg-iAstro cells
LUM_MOUSE	P51885	Lumican	3Tg-iAstro and 4-PBA-treated 3Tg-iAstro cells
TENA_MOUSE	Q80YX1-2	Isoform 2 of Tenascin	3Tg-iAstro and 4-PBA-treated 3Tg-iAstro cells
TSP1_MOUSE	P35441	Thrombospondin-1	3Tg-iAstro and 4-PBA-treated 3Tg-iAstro cells
PZP_MOUSE	Q61838	Pregnancy zone protein	3Tg-iAstro and 4-PBA-treated 3Tg-iAstro cells
PLMN_MOUSE	P20918	Plasminogen	3Tg-iAstro and 4-PBA-treated 3Tg-iAstro cells
ACTB_MOUSE	P60710	Actin, cytoplasmic 1	3Tg-iAstro and 4-PBA-treated 3Tg-iAstro cells
APOA1_MOUSE	Q00623	Apolipoprotein A-I	3Tg-iAstro and 4-PBA-treated 3Tg-iAstro cells
ITIH2_MOUSE	Q61703	Inter-alpha-trypsin inhibitor heavy chain H2	3Tg-iAstro and 4-PBA-treated 3Tg-iAstro cells
K2C1_MOUSE	P04104	Keratin, type II cytoskeletal 1	3Tg-iAstro and 4-PBA-treated 3Tg-iAstro cells
MUG1_MOUSE	P28665	Murinoglobulin-1	3Tg-iAstro and 4-PBA-treated 3Tg-iAstro cells
PEDF_MOUSE	P97298	Pigment epithelium-derived factor	3Tg-iAstro and 4-PBA-treated 3Tg-iAstro cells
RS27A_MOUSE	P62983	Ubiquitin-40S ribosomal protein S27a	3Tg-iAstro and 4-PBA-treated 3Tg-iAstro cells
TRFL_MOUSE	P08071	Lactotransferrin	3Tg-iAstro and 4-PBA-treated 3Tg-iAstro cells
CFAB_MOUSE	P04186	Complement factor B	3Tg-iAstro and 4-PBA-treated 3Tg-iAstro cells
K1C15_MOUSE	Q61414	Keratin, type I cytoskeletal 15	3Tg-iAstro and 4-PBA-treated 3Tg-iAstro cells
COCA1_MOUSE	Q60847-2	Isoform 2 of Collagen alpha-1(XII) chain	3Tg-iAstro and 4-PBA-treated 3Tg-iAstro cells
PAI1_MOUSE	P22777	Plasminogen activator inhibitor 1	3Tg-iAstro and 4-PBA-treated 3Tg-iAstro cells
ACTN4_MOUSE	P57780	Alpha-actinin-4	3Tg-iAstro and 4-PBA-treated 3Tg-iAstro cells
HBB1_MOUSE	P02088	Hemoglobin subunit beta-1	3Tg-iAstro and 4-PBA-treated 3Tg-iAstro cells
NRK_MOUSE	Q9R0G8	Nik-related protein kinase	Only in 3Tg-iAstro cells

K2C75_MOUSE	Q8BGZ7	Keratin, type II cytoskeletal 75	Only in 3Tg-iAstro cells
NRAP_MOUSE	Q80XB4	Nebulin-related-anchoring protein	Only in 3Tg-iAstro cells
VNN3_MOUSE	Q9QZ25	Vascular non-inflammatory molecule 3	Only in 3Tg-iAstro cells
FA92A_MOUSE	Q8BP22	Protein FAM92A	Only in 3Tg-iAstro cells
RC3H2_MOUSE	P0C090	Roquin-2	Only in 3Tg-iAstro cells
TTHY_MOUSE	P07309	Transthyretin (Prealbumin)	Only in 3Tg-iAstro cells
ITIH4_MOUSE	A6X935	Inter alpha-trypsin inhibitor, heavy chain 4	Only in 3Tg-iAstro cells
NUCB1_MOUSE	Q02819	Nucleobindin-1 (CALNUC)	Only in 3Tg-iAstro cells
TRFE_MOUSE	Q921I1	Serotransferrin	Only in 3Tg-iAstro cells
ARAP3_MOUSE	Q8R5G7	Arf-GAP with Rho-GAP domain, ANK repeat and PH domain-containing protein 3	Only in 3Tg-iAstro cells
HBB2_MOUSE	P02089	Hemoglobin subunit beta-2	Only in 3Tg-iAstro cells
TPM4_MOUSE	Q6IRU2	Tropomyosin alpha-4 chain	Only in 3Tg-iAstro cells
K2C1B_MOUSE	Q6IFZ6	Keratin, type II cytoskeletal 1b	Only in 3Tg-iAstro cells
ACTG_MOUSE	P63260	Actin, cytoplasmic 2	Only in 3Tg-iAstro cells
KNG1_MOUSE	O08677	Kininogen-1	Only in 3Tg-iAstro cells
PR7C1_MOUSE	Q9CRB5	Prolactin-7C1	Only in 3Tg-iAstro cells
ARF5_MOUSE	P84084	ADP-ribosylation factor 5	Only in 3Tg-iAstro cells
COF1_MOUSE	P18760	Cofilin-1	Only in 3Tg-iAstro cells
TREA_MOUSE	Q9JLT2	Trehalase	Only in 3Tg-iAstro cells
FABPH_MOUSE	P11404	Fatty acid-binding protein, heart	Only in 3Tg-iAstro cells
TRAP1_MOUSE	Q9CQN1	Heat shock protein 75 kDa, mitochondrial	Only in 3Tg-iAstro cells
TPM2_MOUSE	P58774	Tropomyosin beta chain	Only in 3Tg-iAstro cells
STAG1_MOUSE	Q9D3E6	Cohesin subunit SA-1	Only in 3Tg-iAstro cells
K1C13_MOUSE	P08730	Keratin, type I cytoskeletal 13	Only in 3Tg-iAstro cells
PLXD1_MOUSE	Q3UH93	Plexin-D1	Only in 3Tg-iAstro cells
B4GT1_MOUSE	P15535	Beta-1,4-galactosyltransferase 1	Only in 3Tg-iAstro cells
NEST_MOUSE	Q6P5H2	Nestin	Only in 3Tg-iAstro cells
FABP4_MOUSE	P04117	Fatty acid-binding protein, adipocyte	Only in 3Tg-iAstro cells
PON1_MOUSE	P52430	Serum paraoxonase/arylesterase 1	Only in 3Tg-iAstro cells
LSM4_MOUSE	Q9QXA5	U6 snRNA-associated Sm-like protein LSm4	Only in 3Tg-iAstro cells
XDH_MOUSE	Q00519	Xanthine dehydrogenase/oxidase	Only in 3Tg-iAstro cells
FA8_MOUSE	Q06194	Coagulation factor VIII	Only in 3Tg-iAstro cells
ACTBL_MOUSE	Q8BFZ3	Beta-actin-like protein 2	Only in 3Tg-iAstro cells
NID2_MOUSE	O88322	Nidogen-2	Only in 3Tg-iAstro cells
K1C16_MOUSE	Q9Z2K1	Keratin, type I cytoskeletal 16	Only in 3Tg-iAstro cells

SPA3M_MOUSE	Q03734	Serine protease inhibitor A3M	Only in 3Tg-iAstro cells
ACTA_MOUSE	P62737	Actin, aortic smooth muscle	Only in 3Tg-iAstro cells
CO1A1_MOUSE	P11087-2	Isoform 2 of Collagen alpha-1(I) chain	Only in 4-PBA-treated 3Tg-iAstro cells
CO1A2_MOUSE	Q01149	Collagen alpha-2(I) chain	Only in 4-PBA-treated 3Tg-iAstro cells
GELS_MOUSE	P13020-2	Isoform 2 of Gelsolin	Only in 4-PBA-treated 3Tg-iAstro cells
PGS1_MOUSE	P28653	Biglycan	Only in 4-PBA-treated 3Tg-iAstro cells
FBLN5_MOUSE	Q9WVH9	Fibulin-5	Only in 4-PBA-treated 3Tg-iAstro cells
CATB_MOUSE	P10605	Cathepsin B	Only in 4-PBA-treated 3Tg-iAstro cells
CYTC_MOUSE	P21460	Cystatin-C	Only in 4-PBA-treated 3Tg-iAstro cells
K22E_MOUSE	Q3TTY5	Keratin, type II cytoskeletal 2 epidermal	Only in 4-PBA-treated 3Tg-iAstro cells
LEG1_MOUSE	P16045	Galectin-1	Only in 4-PBA-treated 3Tg-iAstro cells
B2MG_MOUSE	P01887	Beta-2-microglobulin	Only in 4-PBA-treated 3Tg-iAstro cells
ABHD8_MOUSE	Q8R0P8	Protein ABHD8	Only in 4-PBA-treated 3Tg-iAstro cells
TAGL_MOUSE	P37804	Transgelin	Only in 4-PBA-treated 3Tg-iAstro cells
CO3A1_MOUSE	P08121	Collagen alpha-1(III) chain	Only in 4-PBA-treated 3Tg-iAstro cells
K2C8_MOUSE	P11679	Keratin, type II cytoskeletal 8	Only in 4-PBA-treated 3Tg-iAstro cells
CLUS_MOUSE	Q06890	Clusterin	Only in 4-PBA-treated 3Tg-iAstro cells
CC112_MOUSE	A0AUP1	Coiled-coil domain-containing protein 112	Only in 4-PBA-treated 3Tg-iAstro cells
K2C5_MOUSE	Q922U2	Keratin, type II cytoskeletal 5	Only in 4-PBA-treated 3Tg-iAstro cells
S10A6_MOUSE	P14069	Protein S100-A6	Only in 4-PBA-treated 3Tg-iAstro cells
CO6A1_MOUSE	Q04857	Collagen alpha-1(VI) chain	Only in 4-PBA-treated 3Tg-iAstro cells
LDHC_MOUSE	P00342	L-lactate dehydrogenase C chain	Only in 4-PBA-treated 3Tg-iAstro cells
ENOA_MOUSE	P17182	Alpha-enolase	Only in 4-PBA-treated 3Tg-iAstro cells
MOES_MOUSE	P26041	Moesin	Only in 4-PBA-treated 3Tg-iAstro cells
VNN1_MOUSE	Q9Z0K8	Pantetheinase	Only in 4-PBA-treated 3Tg-iAstro cells
EF1A2_MOUSE	P62631	Elongation factor 1-alpha 2	Only in 4-PBA-treated 3Tg-iAstro cells
G3PT_MOUSE	Q64467	Glyceraldehyde-3-phosphate dehydrogenase, testis-specific	Only in 4-PBA-treated 3Tg-iAstro cells
COF2_MOUSE	P45591	Cofilin-2	Only in 4-PBA-treated 3Tg-iAstro cells
FMOD_MOUSE	P50608	Fibromodulin	Only in 4-PBA-treated 3Tg-iAstro cells
ECM1_MOUSE	Q61508	Extracellular matrix protein 1	Only in 4-PBA-treated 3Tg-iAstro cells
PRDX1_MOUSE	P35700	Peroxiredoxin-1	Only in 4-PBA-treated 3Tg-iAstro cells
S23IP_MOUSE	Q6NZC7	SEC23-interacting protein	Only in 4-PBA-treated 3Tg-iAstro cells
APOE_MOUSE	P08226	Apolipoprotein E	Only in 4-PBA-treated 3Tg-iAstro cells
TPM1_MOUSE	P58771	Tropomyosin alpha-1 chain	Only in 4-PBA-treated 3Tg-iAstro cells
IGFN1_MOUSE	Q3KNY0	Immunoglobulin-like and fibronectin type III domain-containing protein 1	Only in 4-PBA-treated 3Tg-iAstro cells
PROF1_MOUSE	P62962	Profilin-1	Only in 4-PBA-treated 3Tg-iAstro cells
CATL1_MOUSE	P06797	Cathepsin L1	Only in 4-PBA-treated 3Tg-iAstro cells
LYOX_MOUSE	P28301	Protein-lysine 6-oxidase	Only in 4-PBA-treated 3Tg-iAstro cells
AMPH_MOUSE	Q7TQF7	Amphiphysin	Only in 4-PBA-treated 3Tg-iAstro cells

CALM1_MOUSE	P0DP26	Calmodulin-1	Only in 4-PBA-treated 3Tg-iAstro cells
ALDOA_MOUSE	P05064	Fructose-bisphosphate aldolase A	Only in 4-PBA-treated 3Tg-iAstro cells
CCL2_MOUSE	P10148	C-C motif chemokine 2	Only in 4-PBA-treated 3Tg-iAstro cells
FLNA_MOUSE	Q8BTM8	Filamin-A	Only in 4-PBA-treated 3Tg-iAstro cells
APOB_MOUSE	E9Q414	Apolipoprotein B-100	Only in 4-PBA-treated 3Tg-iAstro cells
HBA_MOUSE	P01942	Hemoglobin subunit alpha	Only in 4-PBA-treated 3Tg-iAstro cells
PSMD1_MOUSE	Q3TXS7	26S proteasome non-ATPase regulatory subunit 1	Only in 4-PBA-treated 3Tg-iAstro cells
PIAS2_MOUSE	Q8C5D8-2	Isoform 2 of E3 SUMO-protein ligase PIAS2	Only in 4-PBA-treated 3Tg-iAstro cells
RYR2_MOUSE	E9Q401	Ryanodine receptor 2	Only in 4-PBA-treated 3Tg-iAstro cells
SODE_MOUSE	O09164	Extracellular superoxide dismutase [Cu-Zn]	Only in 4-PBA-treated 3Tg-iAstro cells
TXK_MOUSE	P42682-2	Isoform 2 of Tyrosine-protein kinase TXK	Only in 4-PBA-treated 3Tg-iAstro cells
POSTN_MOUSE	Q62009-2	Isoform 2 of Periostin	Only in 4-PBA-treated 3Tg-iAstro cells
TAGL2_MOUSE	Q9WVA4	Transgelin-2	Only in 4-PBA-treated 3Tg-iAstro cells
DJC11_MOUSE	Q5U458	DnaJ homolog subfamily C member 11	Only in 4-PBA-treated 3Tg-iAstro cells
PMS2_MOUSE	P54279	Mismatch repair endonuclease PMS2	Only in 4-PBA-treated 3Tg-iAstro cells
CAB39_MOUSE	Q06138	Calcium-binding protein 39	Only in 4-PBA-treated 3Tg-iAstro cells
MUC4_MOUSE	Q8JZM8	Mucin-4	Only in 4-PBA-treated 3Tg-iAstro cells
ABCBA_MOUSE	Q9JI39	ATP-binding cassette sub-family B member 10, mitochondrial	Only in 4-PBA-treated 3Tg-iAstro cells
IQEC2_MOUSE	Q5DU25	IQ motif and SEC7 domain-containing protein 2	Only in 4-PBA-treated 3Tg-iAstro cells
