# Supplementary figures and images for: Protein synthesis inhibition and loss of homeostatic functions in astrocytes from an Alzheimer’s disease mouse model: a role for ER-mitochondria interaction
Source: Cell Death Dis. 2022 Oct 18;13(10):878. doi: 10.1038/s41419-022-05324-4 (PMC9579125; doi:10.1038/s41419-022-05324-4)

Fig. 1

**b**

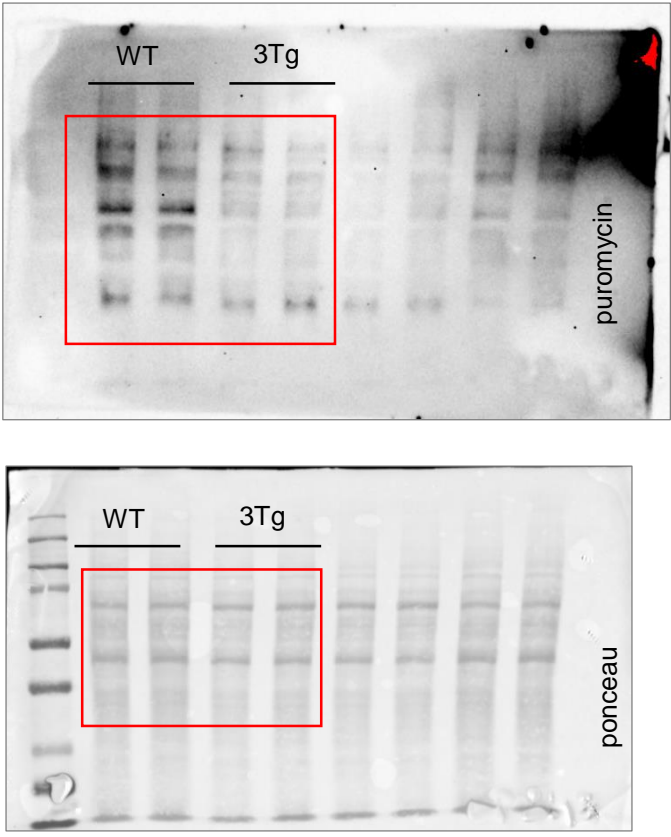

Fig. 2

a

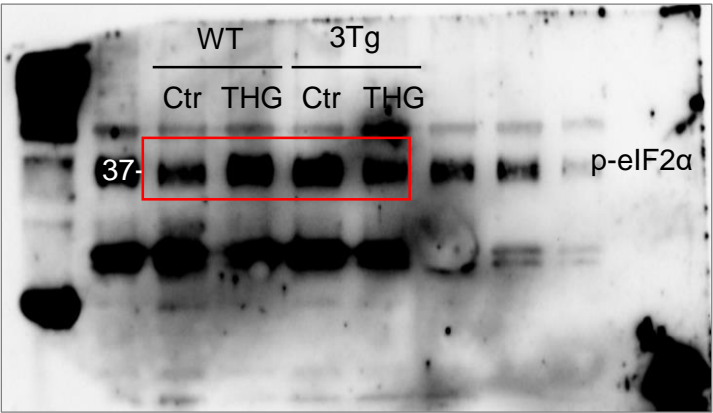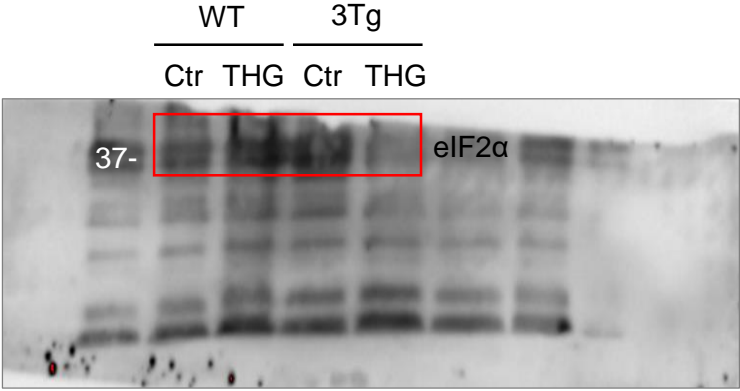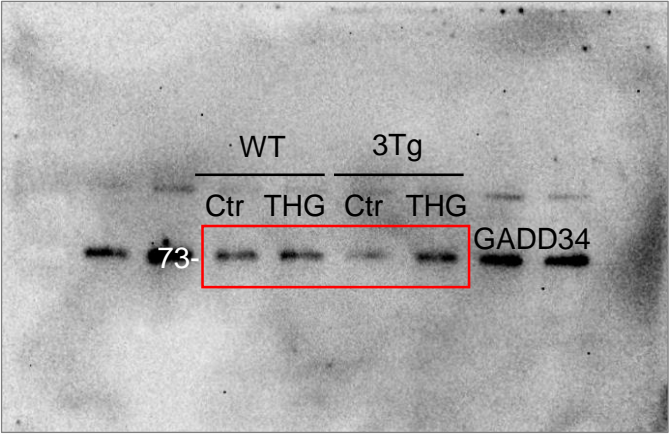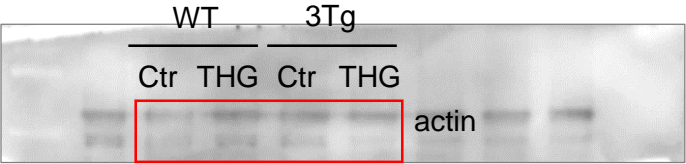

Fig. 2

**b**

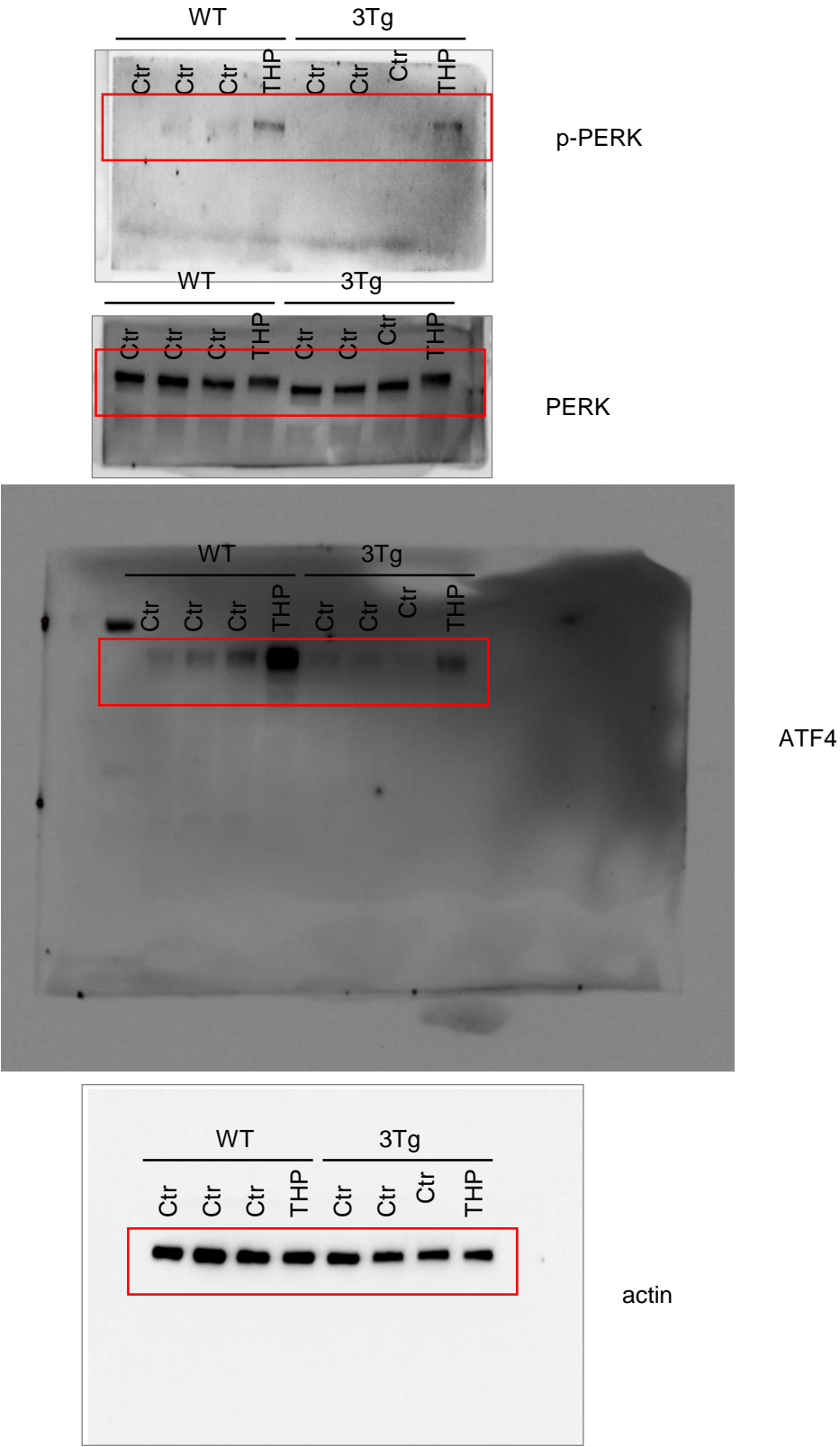

Fig. 4

**a**

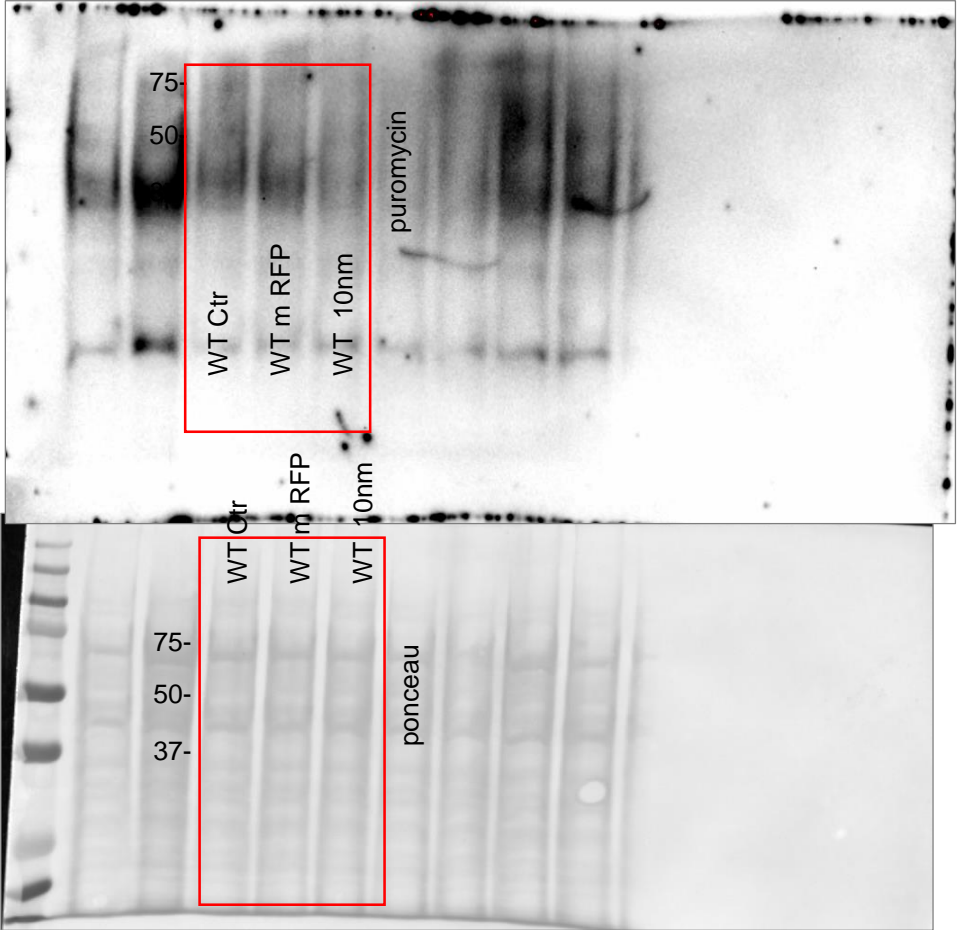

**b**

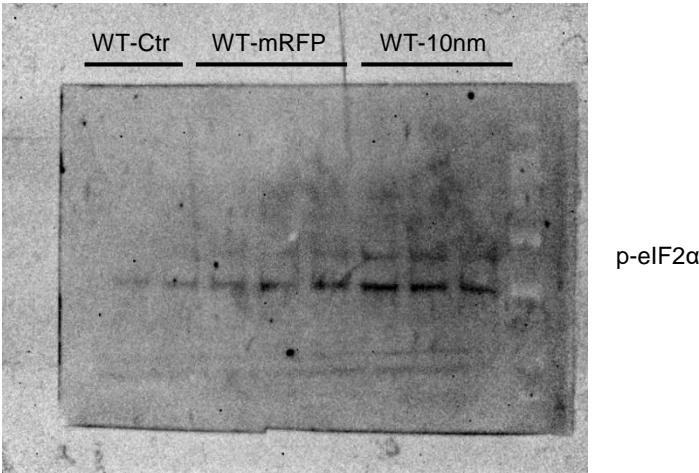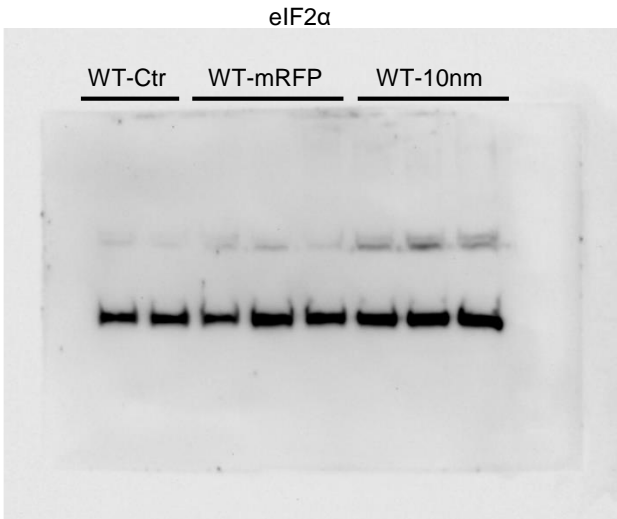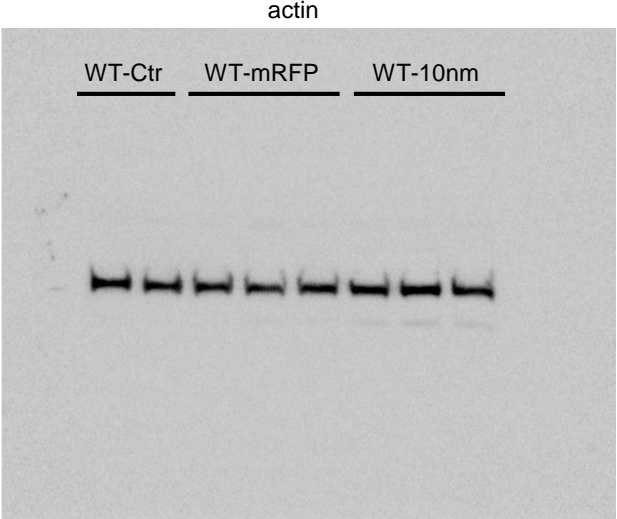

Fig. 5

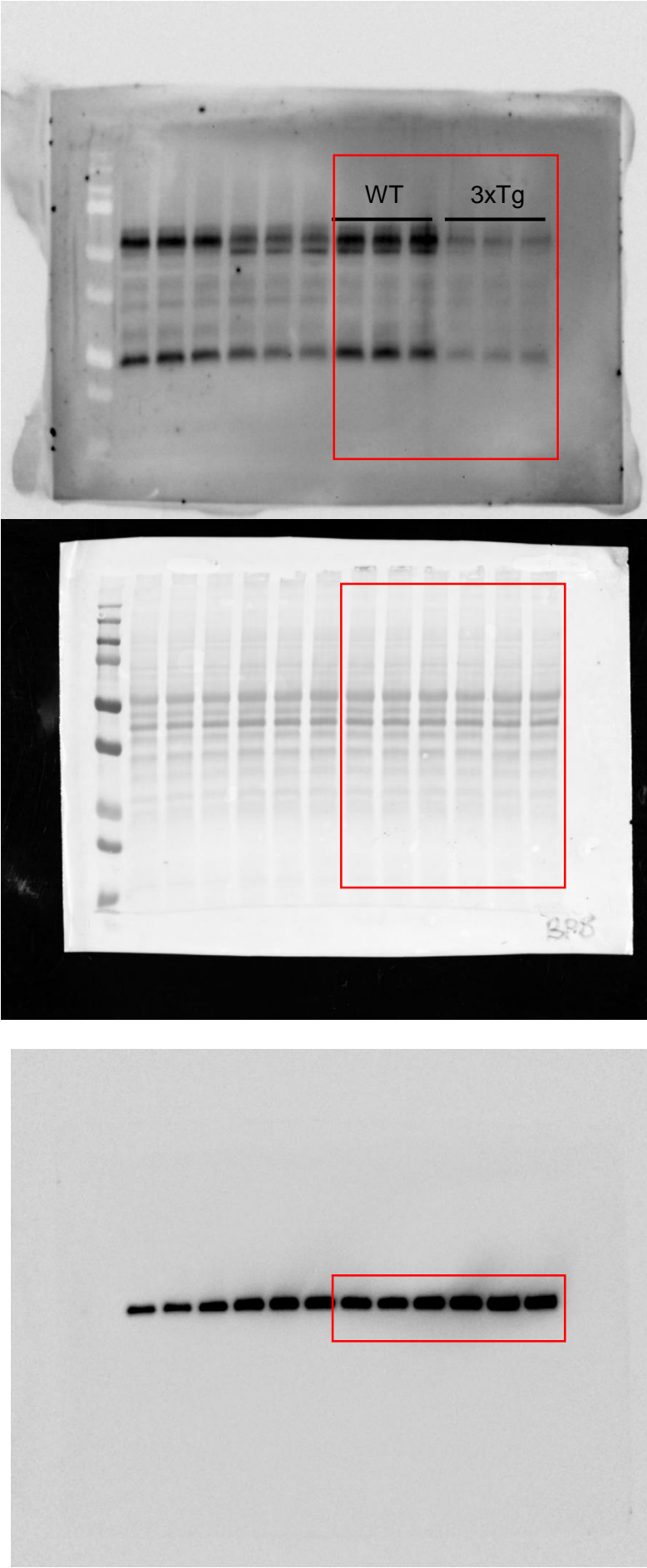

Fig. 6

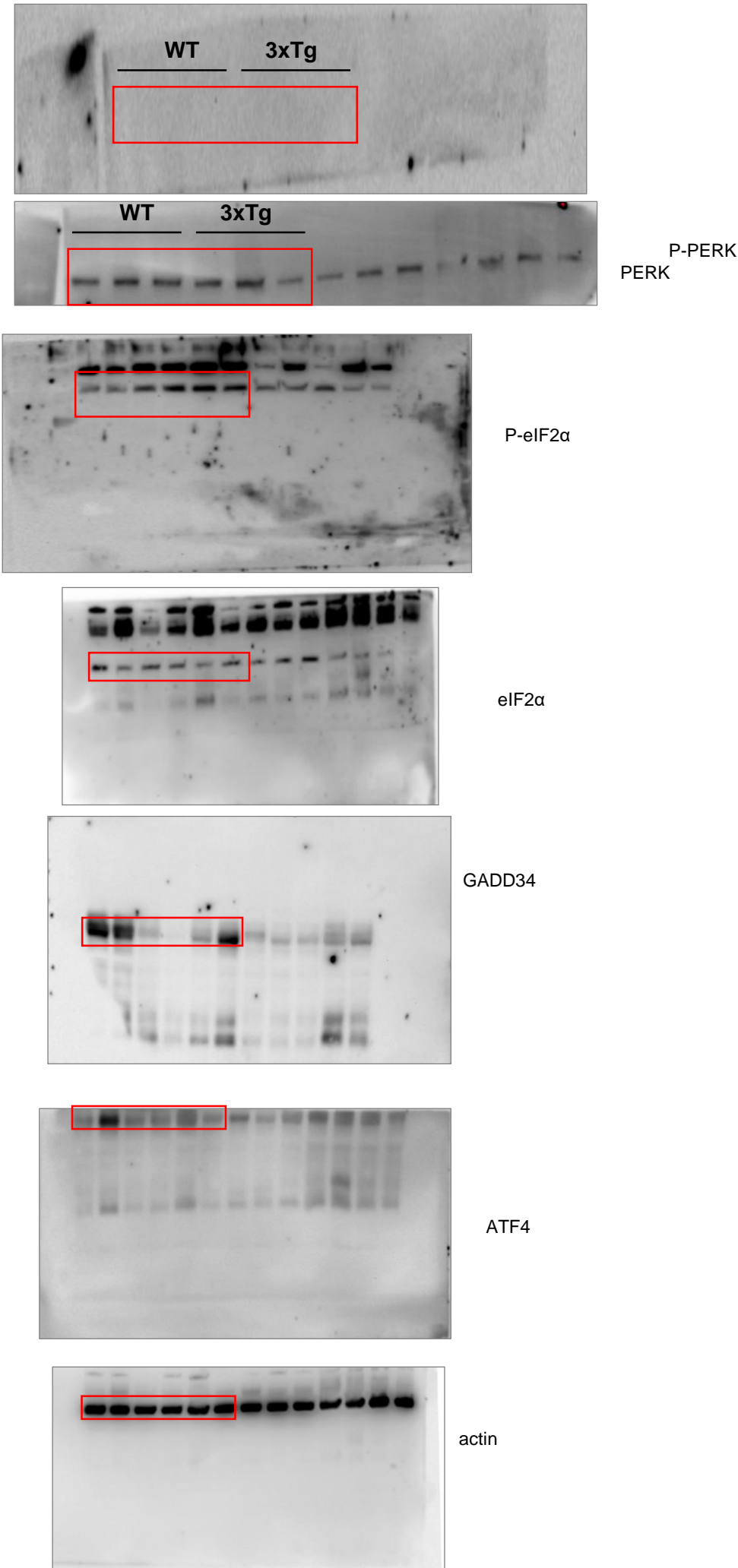

Fig. 7

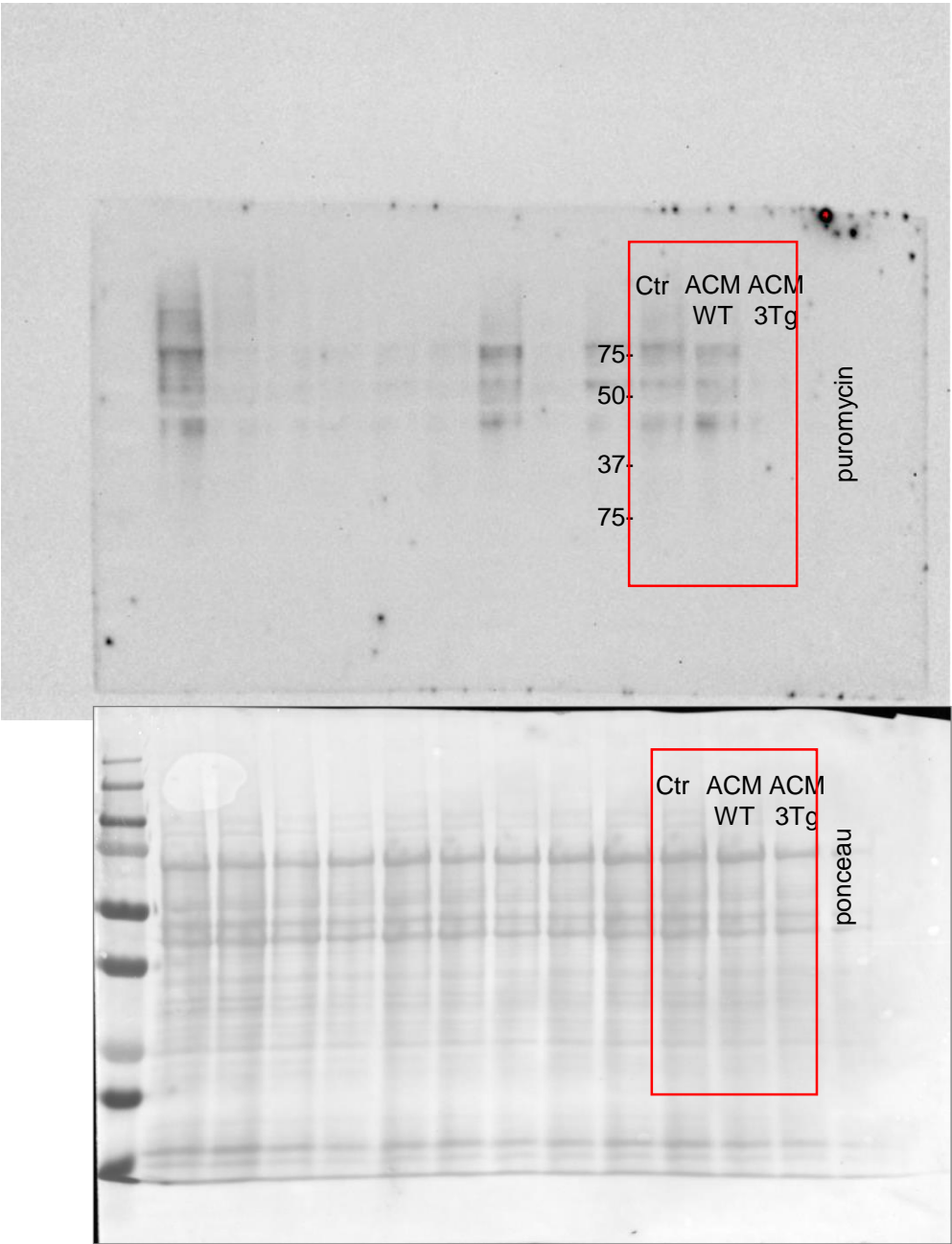

Fig. 9

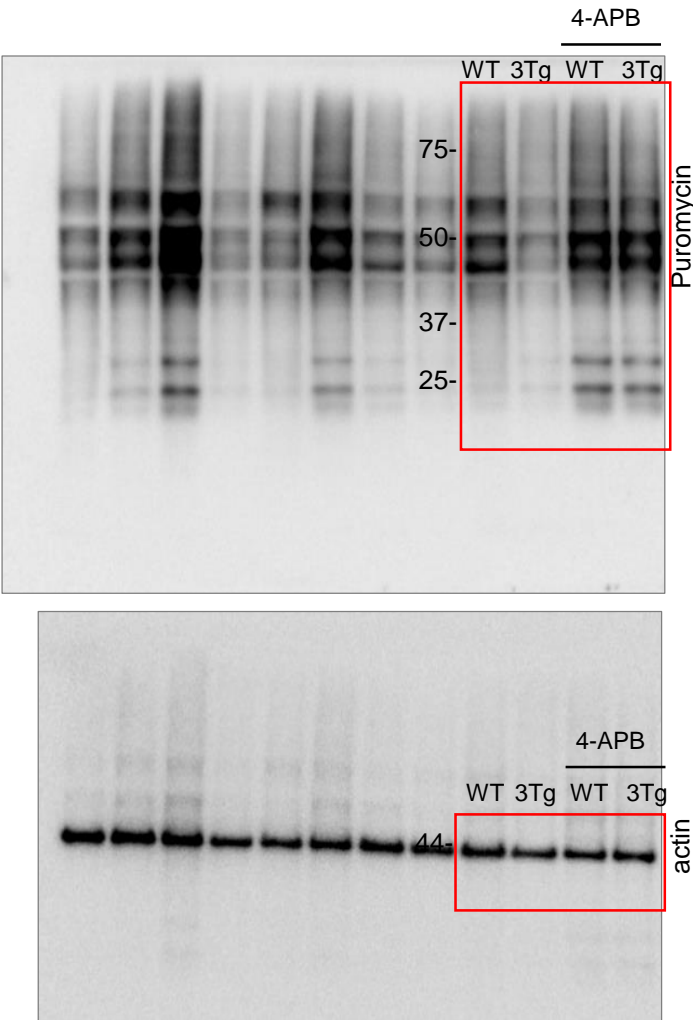

Fig 9

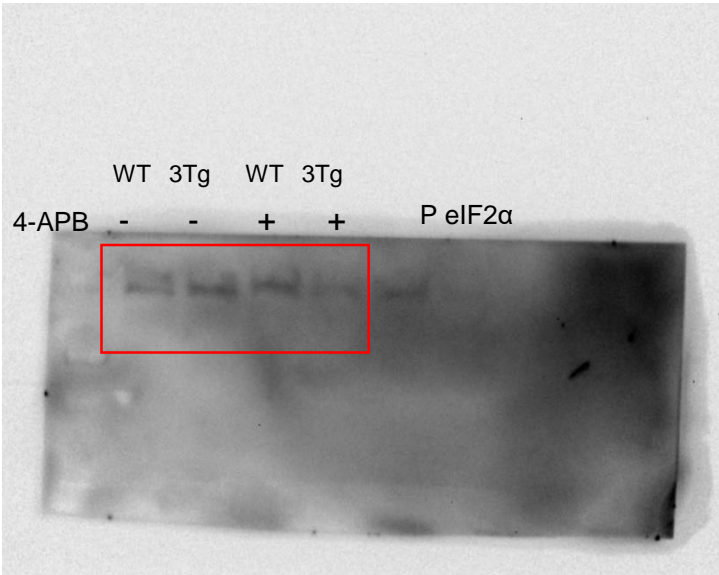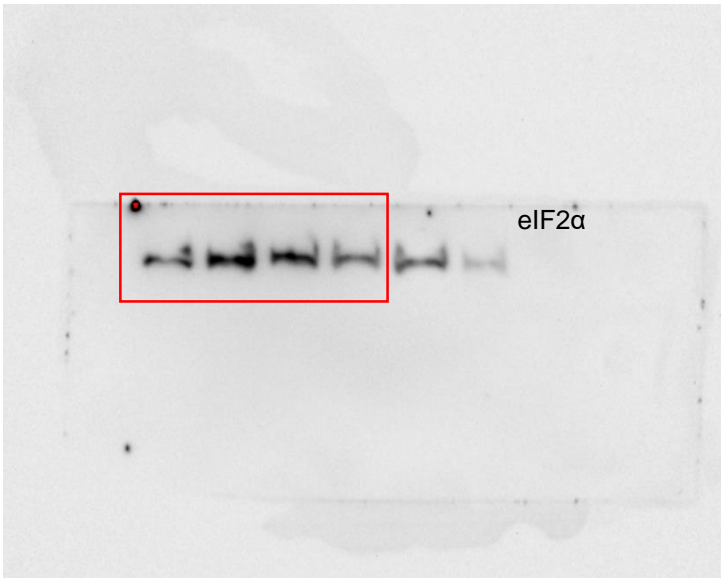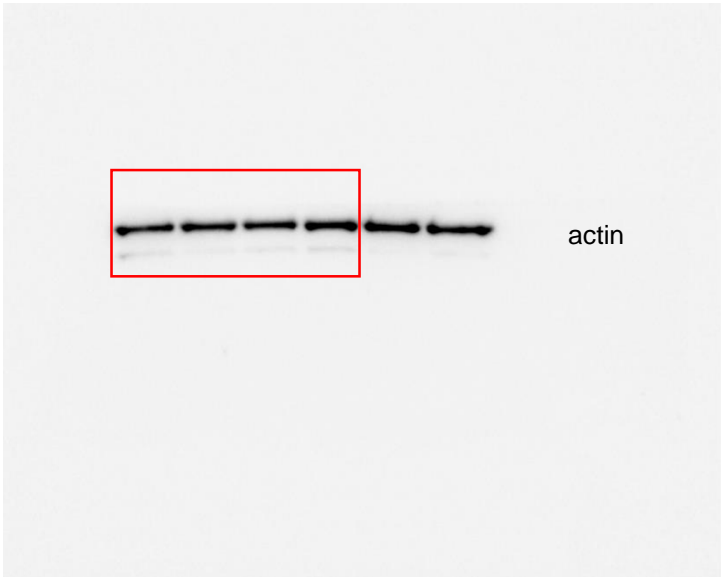

Supplement: Supplementary file 7 — Supplemental Meterial [file 41419_2022_5324_MOESM7_ESM.pdf]
